# Primary health care facilities capacity gaps regarding diagnosis, treatment and knowledge of schistosomiasis among healthcare workers in North-western Tanzania: a call to strengthen the horizontal system

**DOI:** 10.1186/s12913-021-06531-z

**Published:** 2021-05-30

**Authors:** Humphrey Deogratias Mazigo, Cecilia Uisso, Paul Kazyoba, Upendo J. Mwingira

**Affiliations:** 1 Department of Medical Parasitology, School of Medicine, Catholic University of Health and Allied Sciences, P.O. Box 1464, Mwanza, Tanzania; 2 National Neglected Tropical Diseases Control Programme, National Institute for Medical Research, 3 Barack Obama Drive, P.O. Box 9653, 11101 Dar-Es-Salaam, Tanzania; 3 National Institute for Medical Research, 3 Barack Obama Drive, P.O. Box 9653, 11101 Dar-Es-Salaam, Tanzania; 4 RTI International, 701 13th Street NW, 20005 Washington, DC USA

**Keywords:** Primary health care system, Capacity gaps, Schistosomiasis, *Schistosoma haematobium*, *Schistosoma mansoni*, Tanzania

## Abstract

**Background:**

The World Health Organization (WHO) calls for schistosomiasis endemic countries to integrate schistosomiasis control measures into the primary health care (PHC) services; however, in Tanzania, little is known about the capacity of the primary health care system to assume this role. The objective of this study was to assess the capacity of the primary health care system to diagnose and treat schistosomiasis in endemic regions of north-western Tanzania.

**Methods:**

A total of 80 randomly-selected primary health care facilities located in the Uyui, Geita and Ukerewe districts of North-western Tanzania participated in the study. At each facility, the in-charge clinician, or any other healthcare worker appointed by the in-charge clinician, participated in the questionnaire survey. A quantitative questionnaire installed in a Data Tool Kit software was used to collect data. Healthcare workers working at various stations (laboratory, pharmacy, data clerks, outpatient section) were interviewed. The questionnaire collected information related to healthcare workers’ knowledge about urogenital and intestinal schistosomiasis symptoms, human and material resources, laboratory services, data capture, and anti-schistosomiasis treatment availability.

**Results:**

A total of 80 healthcare workers were interviewed. Bloody stool (78.3 %) and haematuria (98.7 %) were the most common symptoms of intestinal and urogenital schistosomiasis mentioned by healthcare workers. Knowledge on the chronic symptoms such as hepatosplenomegaly and hematemesis for intestinal schistosomiasis, and oliguria and dysuria for urogenital schistosomiasis, were inadequate. Laboratory services were only available in 33.8 % (27/80) of the health facilities and direct wet preparation was the most common diagnostic technique used for both urine and stool samples. All healthcare workers knew that praziquantel was the drug of choice for the treatment of schistosomiasis and the drug was available in 91.3 % (73/80) of the health facilities.

**Conclusions:**

The capacity of the primary health care facilities included in the current study is inadequate in terms of diagnosis, treatment, reporting and healthcare workers’ knowledge of schistosomiasis. Thus, the integration of schistosomiasis control activities into the primary healthcare system requires these gaps to be addressed.

**Supplementary Information:**

The online version contains supplementary material available at 10.1186/s12913-021-06531-z.

## Background

Human schistosomiasis is a parasitic disease which is highly prevalent in sub-Saharan Africa (SSA) and is mainly caused by *Schistosoma mansoni* and *Schistosoma haematobium* [1]. *Schistosoma mansoni* causes an intestinal form of the disease, whereas *Schistosoma haematobium* causes the urogenital form of the disease affecting the genito-urinary system. Of the over 200 million cases of the disease which occur worldwide, 93-95 % occur in SSA [1].Tanzania harbours the highest number of schistosomiasis cases after Nigeria [2, 3]. Because of the wide distribution of schistosomiasis, the entire Tanzanian population of approximately 60 million people remains at risk of the disease, and in 2012 it was estimated that 52 % of the population was infected [2, 4]. Both *S. mansoni* and *S. haematobium* are highly endemic and prevalent among communities living along the southern end of the Lake Victoria basin and its islands in the North-western region [5, 6].

In 2020, the World Health Organization (WHO) announced its new goals and vision for controlling and eliminating a number of neglected tropical diseases (NTDs) including schistosomiasis [7]. To achieve this goal, the main strategy recommended specifically for schistosomiasis is periodic preventive chemotherapy using the anti-schistosomal drug praziquantel (PZQ). The aim is to prevent development of morbidity and reduction of infection prevalence [7]. However, the sustainability of this vertical approach remains questionable due to high re-infection rates, high cost, poor coverage and the difficulty of sustaining this intervention over a long period [2]. To achieve the elimination goals, this vertical approach needs to be supplemented with a horizontal approach which will focus on integrating the control measures into the Primary Health Care (PHC) system in order to reach remote rural populations suffering from the disease, populations that are often not reached by mass drug administration (MDA) [8]. In Tanzania, this situation is exacerbated by the decision to carry out targeted chemotherapy [9], addressing only school aged children, therefore reducing the coverage of the infected population [9].

The horizontal approach was recommended by WHO in 1991[8]. An essential aspect of this approach is the clinical care of patients who visit health care facilities with complaints related to infection with schistosomiasis (passive detection) [10]. The disease management at PHC includes all aspects of management i.e. prevention, diagnosis and treatment [10]. However, integrating these measures into the PHC system requires healthcare workers who can recognize the main symptoms of the infection, diagnose the condition, and prescribe praziquantel when appropriate [8, 11]. Also, the use of sensitive diagnostic tests at the PHC level is recommended to expand the differential diagnosis [8, 11]. Previous studies have shown that the integration of schistosomiasis control measures into routine PHC services significantly reduced the prevalence and intensity of schistosomiasis infection [12, 13]. However, many of the schistosomiasis endemic countries in SSA have failed to integrate schistosomiasis intervention measures into the PHC services due to non-availability of drugs, diagnostic tools and poor knowledge of PHC staff [14, 15]. Cumulatively, the capacity of the PHC facilities to integrate schistosomiasis intervention measures such as diagnosis and treatment using PZQ remains unknown in many of the SSA countries, including Tanzania [14–16].

In Tanzania, the PHC is the major entry point into the healthcare system used by the majority of rural communities [17]. The PHC was established in 1967 and further expanded through the decentralization Act in 1972 which led to the development of numerous health facilities in rural areas [18]. The PHC system is managed by the local government administration and is comprised of dispensaries at village/ward levels (serving three to five villages) with an estimated population coverage of 10,000 people [18]. The health centres are also part of PHC and serve as a referral level for the dispensaries; they provide a broader range of services than the dispensaries and cover an average population of 50,000 people [18]. The health care workers serving the PHC system include medical doctors and assistant medical doctors (at health centre level) whereas clinical officers, nurses, midwifery nurses and laboratory technicians can be allocated either to dispensaries or health centres [18].

Schistosomiasis is highly endemic in the rural areas of the country, which are mainly served by the PHC facilities (dispensaries and health centres). Thus, understanding the capacity of the PHC system in terms of diagnosis and management of some of the highly endemic diseases such as schistosomiasis cannot be over emphasized. In that context, the current study focused on assessing the capacity of the PHC system to diagnose and treat schistosomiasis in three schistosomiasis endemic districts (Ukerewe, Geita and Uyui) in North-western Tanzania. The study further assessed the knowledge of healthcare workers on the main symptoms and the availability of diagnosis options and treatment of schistosomiasis. The identified gaps can be used to strengthen the PHC system, ensuring a prompt and appropriate response and management of patients with schistosomiasis.

## Methods

### Study area

**Fig. 1:**
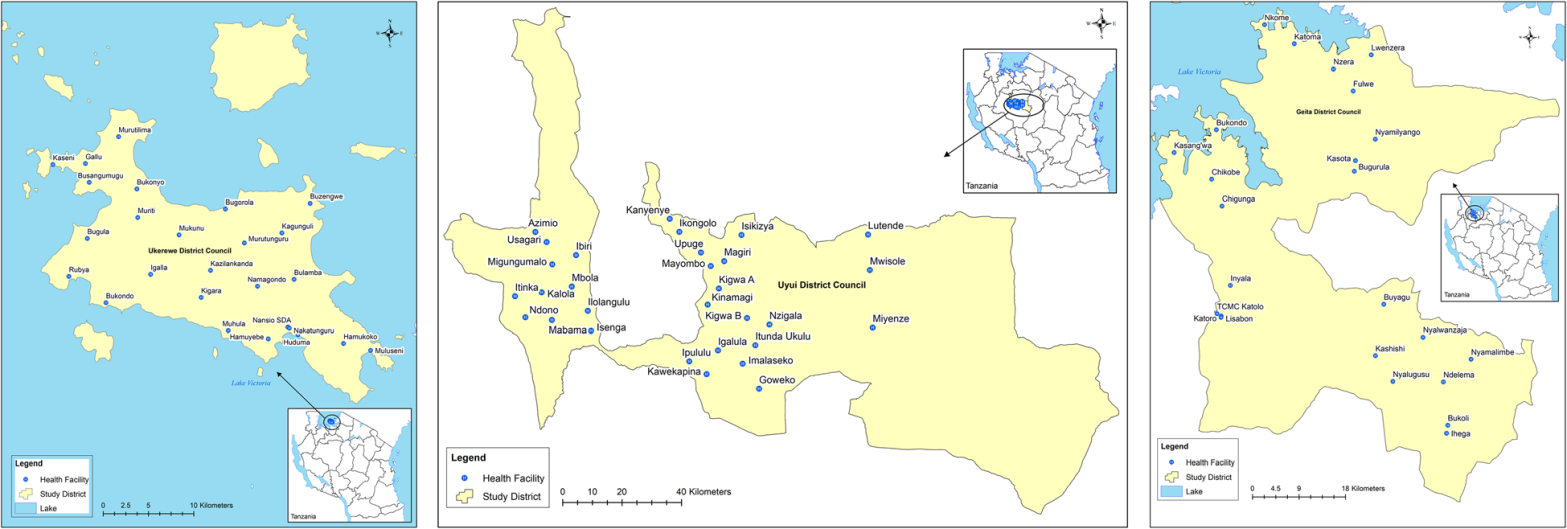
**a**-**c**: Distribution of the health facilities involved in the study from **a**. Ukerewe **b**. Uyui and **c**. Geita districts, north-western Tanzania.

In Tanzania, the health system has five (5) levels: the PHC system (composed of the dispensaries and health centres), district hospital, regional referral hospital and tertiary zonal hospitals. The current study focused on the PHC system in north-western Tanzania and covered selected PHC facilities in three purposively selected districts based on the endemicity of schistosomiasis (Fig. 1). The Ukerewe and Geita districts are located on the southern shorelines of Lake Victoria and are highly endemic for intestinal schistosomiasis [4]. Specifically, Ukerewe is only endemic for intestinal schistosomiasis [4] whereas the Geita district has mixed infections of *S. haematobium* and *S. mansoni*, with communities located along the shoreline of the lake mostly affected by *S. mansoni* infection and those living in the inland areas away from the lake affected by *S. haematobium* [4]. In these two districts, the prevalence of *S. mansoni* ranges from 10 to 80 % [4]. The Uyui district is located on the western part of the Lake Victoria and lies within an area known for *S. haematobium* endemicity [19]. The *S. haematobium* prevalence ranges from 1 to 40 % [3]. In total, the Ukerewe district has one district hospital located at Nansio (capital of the district), four (4) health centres and 32 dispensaries located in various islands on Lake Victoria (district health report). Geita has one district hospital, four (4) health centres and 39 dispensaries, whereas the Uyui district has one district hospital, one health centre and 44 dispensaries. All of the mentioned health facilities are owned by the Tanzanian government.

### Study design

This was a cross-sectional study designed to assess the capacity of the PHC system to diagnose and treat schistosomiasis with particular focus on healthcare workers’ knowledge of symptoms and clinical signs related to schistosomiasis, availability of praziquantel tablets at the time when this study was conducted, available options for diagnosis and treatment of schistosomiasis, availability of laboratory facilities within the health facilities building, and health care workers available at the health facilities. The main inclusion criteria for a health facility were: being in the study area, being a public health facility, serving at least 50 patients/clients per week and being accessible. A total of 124 PHC facilities were in the study area and 80 health facilities were randomly selected for the study (24 from Geita district, 26 from Ukerewe and 29 from Uyui). Most of the selected health facilities were serving communities known to be highly endemic to schistosomiasis [3]. Figure 1 shows the distribution of the health facilities involved in the study. The study was conducted from October-November 2019.

### Data collection

The study adapted a questionnaire which was used by a similar previous study in Burundi [15]. The tool was translated from English to Kiswahili and pre-tested in the Magu district (a different district from the study districts). Corrections were made to questions which were not clear to participants, and questions which had no meaning or did not fit the Tanzanian setting were removed/not included in the final questionnaire. After data collection, back translation was done to allow data entry and analysis. The questionnaire collected the following information (i) type of health facility (ii) knowledge and types of healthcare workers available in each health facility (iii) knowledge of symptom(s) related to both *S. mansoni* and *S. haematobium* (iv) diagnosis (knowledge of how schistosomiasis is diagnosed, availability of laboratory space in the health facility, availability of laboratory protocol and diagnostic tests for *S. haematobium* and *S. mansoni*) (v) treatment (availability, purchasing and supply process (vi) case reports in the database. In each of the health facilities, the in-charge clinician of the health facilities was interviewed. If the in charge was not available, the head of a selected section (pharmacy, laboratory, or nursing) of the health facility was interviewed. The interview was conducted individually (as a one-on-one interview) for each participant within the health facility. In total, 80 health workers were interviewed in each facility. Ten trained graduates with medical doctor degrees who were waiting to join internship were recruited as data collectors/research assistants. All received a two-day training and participated in the pre-testing of the data collection tool and field data collection.

### Data analysis

Data were collected using a Tropical Data Kit Tools with GPS software installed on a mobile phone or tablets [20]. To ensure quality of collected data, all data received each day were reviewed by two people and any errors were communicated with the data collection team before the next round of data collection. At the field sites, at the end of each day, the field team reviewed the collected data and any challenges faced were communicated with the team handling the database. Collected data were downloaded into an excel file (Microsoft, Redmond, US) checked and exported to STATA version 15 (StataCorp, LP, College Station, US). Using the data dictionary, data were coded, cleaned, and analysed. The statistical plan focused mainly on generating frequencies for categorical variables with their 95 % confidence intervals for proportions. Continuous (age, curative visits, etc.) variables were summarised using mean ± standard deviation (SD). If comparison was to be done, continuous variables were compared using either t-test or ANOVA (for more than two variables).

## Results

### Characteristics of the health facilities involved in the study

A total of 80 health facilities were involved in the study, of these, 86 % were dispensaries, 10 were health centres and 1 was a district hospital. Table 1 shows the numbers and types of health facilities involved in the study for each district.

**Table 1:** Types of health facilities from each study district

District	Type of health facilities			
	Dispensary	Health centre	District hospital	
Geita	17 (24.6 %)	6 (60 %)		1 (100 %)
Ukerewe	23 (33.3 %)	3 (30 %)		0
Uyui	29 (42 %)	1 (10 %)		0

## Composition of health workers in the health facilities

Table 2 below shows the numbers and types of staff at all the levels of health facilities that participated in the study. Healthcare workers with the highest qualifications such as medical doctors were mainly stationed at the level of health centres while at the dispensary level, the majority were clinical officers and few assistant medical officers. Conversely, enrolled nurses and medical attendants were mainly concentrated more at the level of dispensaries than the health centre. Laboratory technicians were only present in the health facilities which offered medical laboratory services [27 facilities, 17/27 (62.9 %) were dispensaries and 10/27, (37.1 %) health centres]. Other healthcare workers such as pharmacists were mainly stationed at the district hospital and health centres.

**Table 2:** Healthcare workers available at the dispensaries and health center visited

Healthcare worker type	Type of primary PHC facility	
	**Dispensary**	**Health centre**
Medical doctors	0 (0 %)	4(7.6 %)
Clinical officers/assistant medical officers	37(21 %)	8(15.4 %)
Enrolled nurses	60(34.1 %)	10(19.2 %)
Midwives	16(9.1 %)	5(9.6 %)
Medical Laboratory technicians	17(9.6 %)	10(19.2 %)
Pharmacists	1(0.5 %)	5(9.6 %)
Medical attendants	45(25.5 %)	10(19.2 %)
**Total**	176 (100 %)	52 (100 %)

### Healthcare workers’ knowledge about schistosomiasis

The knowledge of healthcare workers about urogenital schistosomiasis related symptoms and clinical signs was assessed. Table 3 summarizes the responses provided by the interviewed healthcare workers. Overall, among the interviewed healthcare workers, 96.2 % knowing the symptoms related to *S. haematobium*, with the most commonly mentioned symptom being haematuria (98.7 %). Knowledge about other *S. haematobium* related symptoms such as oliguria, dysuria/pain during urination and pollakiuria was very low among interviewed healthcare workers. Nonspecific symptoms mentioned related to *S. haematobium* were abdominal pain, joint pain, back pain, pus in urine and skin rashes.

**Table 3:** Symptoms related to *S. haematobium* as mentioned by interviewed healthcare workers.

Variable	n	%
Know about any symptoms related to *S. haematobium*		
Yes	77	96.3
No	3	3.7
Macroscopic haematuria		
Yes	76	98.7
No	1	1.3
Pollakiuria		
Yes	5	6.5
No	72	93.5
Dysuria		
Yes	50	64.9
No	27	35.1
Oliguria		
Yes	4	5.2
No	73	94.8
Nephrotic colic		
Yes	20	25.9
No	57	74.0
Anaemia		
Yes	14	18.2
No	63	81.2
Asthenia		
Yes	3	3.9
No	74	96.1

Table 4 summarizes the reported symptoms of *S. mansoni* infection (intestinal schistosomiasis) by interviewed healthcare workers. In general, the knowledge level of healthcare workers was very low, with the major symptoms reported being bloody stool (78.3 %) and abdominal pain (82.6 %). Knowledge about other *S. mansoni* related symptoms such as hepatomegaly, splenomegaly and blood vomiting were also very low. Other non-specific symptoms mentioned included back pain (5), vomiting (1), loss of body weight and headache (1), general body weakness and fever (1), fever and joint pain (1)

**Table 4:** Reported symptoms related to *Schistosoma mansoni* (intestinal schistosomiasis)

Variable	n	%
Know about symptoms related to *S. mansoni* infection		
Yes	69	86.3
No	11	13.7
Diarrhoea		
Yes	22	31.9
No	47	68.1
Bloody stool		
Yes	54	78.3
No	15	21.7
Abdominal pain		
Yes	57	82.6
No	12	17.4
Hepatosplenomegaly		
Yes	18	26.1
No	51	73.9
Oedema		
Yes	4	5.8
No	65	94.2
Ascites		
Yes	20	28.9
No	49	71.0
Haematemesis/blood vomiting		
Yes	25	36.2
No	44	63.8
Anaemia		
Yes	14	20.3
No	55	79.7
Asthenia		
Yes	4	5.8
No	65	94.2

### Laboratory testing available for diagnosis of intestinal and urogenital schistosomiasis

Only 33.8 % (27/80) of health facilities visited had a special room dedicated for laboratory services and had a laboratory technician. Review of the past records indicated that schistosomiasis (both *S. mansoni* and *S. haematobium*) and soil-transmitted helminths (mainly *Ascaris lumbricoides*) were commonly diagnosed.

The most common diagnostic method used was a direct smear technique both for urine and stool samples. Urine reagent dipsticks, which can be used for diagnosis of haematuria for *S. haematobium* infection, were only available in 55.5 % (15/27) of the health facilities. For health facilities which had no laboratory services, patients were either treated symptomatically without diagnosis or referred to the nearby health facility/private laboratory for diagnosis before treatment. Supplementary Table shows the available laboratory equipment. Stains useful for *S. mansoni* or *S. haematobium* eggs were available in 37 % of the health facilities.

### Drug supply and healthcare workers’ knowledge of the available treatment options for schistosomiasis

Overall, all the interviewed healthcare workers knew that PZQ was the drug of choice and recommended for treatment of schistosomiasis (both *S. mansoni* and *S. haematobium*). However, when asked if there was another drug which can treat schistosomiasis, the following drugs were mentioned ciprofloxacin (2), erythromycin (2), mebendazole (1), metronidazole (2) and vitamin B complex (1). When asked if they remembered the PZQ dose given per kilogram of body weight (Kgbwt), 63.8 % (51/80) reported to know the dosage. However, when asked to mention it, 86.3 % (n = 44/51) mentioned correctly that it was 40 mg/Kgbwt, the remaining mentioned that the recommended dosage was 30 mg/Kgbwt.

PZQ was reported to be available in 91.3 % (73/80) of the health facilities that participated in the study (Table 5), with only one health facility having a stock of 12 tins (each with 500 tablets). Only seven dispensaries did not have PZQ in stock. For the health facilities which did not have PZQ at the time of this study, participants reported that they could expect to spend an average of 26.6 days (95 %CI: 7.2–46.0, range 0-365 days) without having the drug in stock.

**Table 5:** Availability of praziquantel categorised by the type of health facilities

Praziquantel availability	Type of health facilities		
	Dispensary	Health centre	District hospital
Yes	62 (89.9 %)	10 (100 %)	1 (100 %)
No	7 (10.1 %)	0	0

### Schistosomiasis case reporting

On average, 2,125.1 (95 %CI: 568.7-3681.5) curative visits were recorded in the last 12 months in all the health facilities, ranging from 800 − 55,000 curative visits. The district hospital reported an average of 18,845 cases, health centres an average of 7,219 cases and dispensaries an average of 1,144 cases (F = 7.15, *P* < 0.001). The median number of hospital beds was 5 beds (IQR: 3-8.5 beds).

In relation to schistosomiasis case reporting, only 64/80 (80 %) of the health facilities reported that they recorded schistosomiasis cases in the National Health Information System and had running computer systems. However, data were entered in a group called helminths with no species-specific names. The remaining health facilities 16/80 (20 %) used hand written registers to record all the disease cases including schistosomiasis.

## Discussion

The findings of the present study demonstrated that there was a limited capacity of the PHC system to diagnose and manage schistosomiasis cases. Laboratory services were not available in almost two thirds of the health facilities visited and direct wet smear, which lacks sensitivity, was the main technique used for the diagnosis of schistosomiasis. Healthcare workers had limited knowledge of schistosomiasis-related symptoms, especially of chronic symptoms and signs related to both *S. haematobium* and *S. mansoni* infection.

The management of schistosomiasis cases in terms of diagnosis using either recommended laboratory techniques or presumptive diagnosis depends on the presence of knowledgeable healthcare workers [14, 15], who are able to suspect schistosomiasis in patients with related symptoms and transfer them to the laboratory for diagnosis or offer presumptive treatment based on identified symptoms which could be related to schistosomiasis. The present study noted that the knowledge of healthcare workers on schistosomiasis-related symptoms, especially chronic symptoms related to either *S. mansoni* or *S. haematobium*, was inadequate. Most of the healthcare workers reported to know haematuria, an indicator of *S. haematobium* infection [21] and bloody stool, an indication of *S. mansoni* infection [22]. The relationship between either haematuria and *S. haematobium* [21] or *S. mansoni* and bloody in stool is well described elsewhere [23]. However, it is worth noting that, in the tropics, haematuria and blood in stool are not uniquely caused by either *S. haematobium* [21] or *S. mansoni* [24]. They could also be caused by other aetiological agents. Inadequate knowledge of the main symptoms of intestinal schistosomiasis was also noted in Burundi [15]. In contrast, nurses in the Democratic Republic of Congo (DRC) [25] and Mali [16] had a good knowledge of the main symptoms related to *S. mansoni* and *S. haematobium*. Our findings and those of other authors [15] highlight the need for in-service training which can bring added value in strengthening the capacity of the PHC to identify and manage patients with symptoms suggestive of *S. haematobium* and *S. mansoni*. In fact, in Senegal and Mali, in-service training interventions led to improved knowledge of healthcare workers on the main symptoms of urogenital and intestinal schistosomiasis[16, 26]. Improved knowledge led to an increased likelihood that a patient presenting with haematuria and blood in stool received PZQ treatment [26].

Laboratory services form an important component of case management at the PHC level, not only for the diagnosis of schistosomiasis suspected cases but also for the diagnosis of other endemic diseases [27]. In the present study, almost two-thirds of the health facilities did not have laboratory services, with majority of them being dispensaries. Unavailability of laboratory services at PHC sites appears to be a common problem in many of the schistosomiasis endemic countries of SSA [15]. In the DRC, most health centres did not have laboratory services for the diagnosis of schistosomiasis [25]. Unavailability of laboratory equipment and reagents was also noted among primary health facilities in Burundi [15]. While the WHO recommends the use of the urine sedimentation, centrifugation or filtration techniques for the diagnosis of *S. haematobium* in urine and Kato Katz (mostly used for epidemiological surveys) or formal-ether concentration techniques for the diagnosis of *S. mansoni* in stool [27], most of the health facilities which offered laboratory services preferred to use urine reagent dipsticks for *S. haematobium* and the direct wet smear test for *S. mansoni*. Similar findings have been reported by previous studies [15, 26]. In Mali, the extra time needed to perform either the urine filtration or Kato Katz techniques, as compared to direct wet preparation, was mentioned as the main reason for not using these techniques [26]. It is worthwhile noting that urine filtration and Kato Katz techniques have a significantly higher performance than the direct wet preparation, which leaves a proportion of infected individuals undiagnosed [27]. Recently, a Point-of-Care Circulating Cathodic Antigen test, a rapid test which does not require electricity or other reagents, has been introduced for the diagnosis of *S. mansoni* infection [28]. The test is highly sensitive compared to the Kato Katz technique and performs adequately for *S. mansoni* infection but is not reliable for the detection of *S. haematobium* infection [29, 30]. The rapid test is easy to use and uses urine instead of stool samples [28]. However, this rapid test is more expensive than the Kato Katz and therefore its introduction into the PHC system may be impractical as patients may fail to pay the related costs.

We noted the unavailability or the inadequate supply of PZQ in some of the investigated health facilities, which requires prompt action. A similar observation was made by previous studies in SSA [15, 25]. None of the visited health centres in DRC [25] and Burundi [15] had PZQ in stock. In Ghana and Senegal, PZQ was reported to be out of stock in 22.5 % [31] and 25 % [32] of the health facilities respectively. Because of the unavailability of PZQ in the health facilities, patients were referred or asked to seek for the drug at a nearby health facility or from a private pharmacy [25]. The unavailability or inadequate supply of PZQ in health facilities serving highly endemic communities such as those living along the shoreline of Lake Victoria in Tanzania needs an urgent response from the relevant authorities if schistosomiasis elimination remains a priority health agenda. Without adequate PZQ stocks at PHC level facilities, especially those serving highly endemic communities, it will be difficult if not impossible to achieve the 2030 goals of eliminating schistosomiasis in Tanzania. The wide network of the horizontal PHC system, which reaches high risk populations not covered by the MDA, may help to shrink the schistosomiasis map by expanding the treatment coverage and reaching remote populations carrying the highest burdens of the disease.

On the other hand, the findings of this study show that 80 % of the PHC were connected to the National Health Information System (NHIS). Similar findings have been noted elsewhere in SSA [14]. However, data on schistosomiasis were aggregated into one group called “helminth infection” and were not entered using a term related to a specific helminth infection. This challenge has also been noted in skin related NTD infections, in which data related to these diseases are aggregated in one group called “skin diseases” [33]. Data capturing at PHC level remains a significant challenge in SSA where the National Health Insurance Fund (NHIF) or surveillance system is not universally implemented and is too weak to ensure high-quality data collection, analysis and dissemination [34, 35]. Capturing health data related to neglected tropical diseases at PHC level can be used to predict the need, monitor the progress and measure the impact of community-based control interventions such as MDA. Thus, as the world continues to strive to reach the 2030 goals of eliminating schistosomiasis as a public health problem, building resilient health information systems at the PHC level should be an essential part of the process. Building capacity in the areas of data processing, analysis and sharing is highly recommended.

In general, there is a need to strengthen the PHC system in terms of diagnosis and identification of schistosomiasis, as population coverage using the vertical approach is limited and costly [14]. The wide network of the horizontal PHC system, which reaches high risk populations not covered by the MDA, will complement MDA coverage and subsequently lead to the reduction of the prevalence of schistosomiasis [12, 13]. Alternatively, the introduction of sentinel health facilities with improved laboratory services and trained staff in highly endemic areas like Ukerewe and Geita districts will help to improve the management of schistosomiasis cases [25]. In Sudan, the integration of the horizontal PHC based system into the control of schistosomiasis led to a higher population coverage and resulted in significant reduction of the prevalence of both *S. mansoni* and *S. haematobium* infection [36]. To reach the elimination goals, strengthening the capacity of PHC facilities to diagnose and treat schistosomiasis should go hand-in-hand with the improvement of knowledge of PHC workers about schistosomiasis and its management, the implementation of other community-based control measures such as improved water supply and sanitation, snail control and the provision of health education to endemic communities [37].

The current study was not conducted without limitations. The study included only 80 PHC workers out of over 200 healthcare workers working in the primary health facilities located in the study areas. This may affect the generalizability of the findings, especially on the knowledge about schistosomiasis symptoms and its management. However, we believe that the picture given by the visited PHC facilities in relation to schistosomiasis diagnosis, the healthcare workers’ knowledge and the management of schistosomiasis cases would be similar in most of the PHC facilities of North-western Tanzania. In addition, the descriptive cross-sectional nature of the study design could not provide data on causality and allow comparisons of the outcome of interest between health facilities and districts. Nevertheless, the findings from the current study allow the following conclusions and considerations.

## Conclusions

Overall, the capacity of the primary health facilities included in the current study is inadequate in the areas of diagnosis, treatment, reporting and healthcare workers’ knowledge. Inavailability of PZQ and laboratory services in some of the health facilities requires an urgent response from the responsible authorities. In addition, the use of a direct wet smear for the diagnosis of urogenital and intestinal schistosomiasis and the lack of drugs need to be addressed to allow the detection of the majority of schistosomiasis infected persons and to adapt symptoms-based direct treatment. Moreover, the insufficient knowledge of healthcare workers on the chronic morbidities associated with either urogenital or intestinal schistosomiasis affected their capacity to identify chronic cases of the disease and led to denying patients appropriate treatment. This has to be promptly addressed. Overall, to meet the 2030 goals for schistosomiasis elimination and to ensure that no one is left behind, the primary health care facilities’ capacity must be strengthened for the case management of schistosomiasis. This could be relevant for other endemic diseases as well.

## Supplementary Information

**Additional file 1:** **Supplementary table**: Available laboratory equipment in the health facilities offering laboratory services

## Data Availability

The datasets generated and/or analysed during the current study are not publicly available due to the fact authors did not request permission from the institutional review board/ethical committee to share the data file but data files are available from the corresponding author on reasonable request.

## Abbreviations

PPF: Periportal fibrosis
MDA: Mass Drug Administration
SAC: School aged children
PSAC: Pre-school aged children
PZQ: Praziquantel
KK: Kato Katz technique
POC-CCA: Point-of-Care Circulating Cathodic Antigen
GPS: Geographical Positioning System
NIMR: National Institute for Medical Research
NTD: Neglected Tropical Diseases
