# Supplementary material for: Primary health care facilities capacity gaps regarding diagnosis, treatment and knowledge of schistosomiasis among healthcare workers in North-western Tanzania: a call to strengthen the horizontal system
Source: BMC Health Serv Res. 2021 May 30;21:529. doi: 10.1186/s12913-021-06531-z (PMC8165992; doi:10.1186/s12913-021-06531-z)
Supplement: Supplementary file 1 — Additional file 1: Supplementary table: Available laboratory equipment in the health facilities offering laboratory services [file 12913_2021_6531_MOESM1_ESM.docx]

**Supplementary table:** Available laboratory equipment in the health facilities offering laboratory services

| **Equipment** | **n=27** | **%** |
| --- | --- | --- |
| **Equipment and reagents available for diagnosis of S. haematobium** | | |
| **Microscope** | | |
| Yes | 27 | 100 |
| No |  |  |
| **Object and cover slides** | | |
| Yes | 21 | 77.8 |
| No | 6 | 22.2 |
| **Centrifuge** | | |
| Yes | 17 | 62.9 |
| No | 10 | 37.1 |
| **Membrane filters** | | |
| Yes | 5 | 18.5 |
| No | 22 | 81.5 |
| **Filter holders** | | |
| Yes | 4 | 14.8 |
| No | 23 | 85.2 |
| **Syringes** | | |
| Yes | 26 | 96.3 |
| No | 1 | 3.7 |
| **Centrifuge tubes** | | |
| Yes | 16 | 59.3 |
| No | 11 | 40.3 |
| **Iodine (5%)** | | |
| Yes | 10 | 37.0 |
| No | 17 | 62.9 |
| **Urine dipstick** | | |
| Yes | 15 | 55.5 |
| No | 12 | 44.4 |
| **Equipment and reagents available for diagnosis of *S. mansoni*** | | |
| **Object slides** | | |
| Yes | 26 | 96.3 |
| No | 1 | 3.7 |
| **Malachite green** | | |
| Yes | 4 | 14.8 |
| No | 23 | 85.2 |
| **Methylene blue** | | |
| Yes | 22 | 81.5 |
| No | 5 | 18.5 |
| **Glycerine solution 50%** | | |
| Yes | 6 | 22.2 |
| No | 21 | 77.8 |
| **Cellophane** | | |
| Yes | 1 | 3.7 |
| No | 26 | 96.3 |
| **Kato Katz template** |  |  |
| Yes | 0 | 0 |
| No | 27 | 100 |
| **Spatula** | | |
| Yes | 14 | 51.8 |
| No | 13 | 48.2 |
